# Antimicrobial Activity and Phytochemical Screening of *Buchenavia tetraphylla* (Aubl.) R. A. Howard (Combretaceae: Combretoideae)

**DOI:** 10.1100/2012/849302

**Published:** 2012-12-24

**Authors:** Ygor Lucena Cabral de Oliveira, Luís Cláudio Nascimento da Silva, Alexandre Gomes da Silva, Alexandre José Macedo, Janete Magali de Araújo, Maria Tereza dos Santos Correia, Márcia Vanusa da Silva

**Affiliations:** ^1^Laboratório de Produtos Naturais, Departamento de Bioquímica, Centro de Ciências Biológicas, Universidade Federal de Pernambuco, Avenida Professor Moraes Rêgo, s/n Cidade Universitária, 50670-420 Recife, PE, Brazil; ^2^Laboratório de Glicoproteínas, Departamento de Bioquímica, Centro de Ciências Biológicas, Universidade Federal de Pernambuco, Avenida Professor Moraes Rêgo, s/n Cidade Universitária, 50670-420 Recife, PE, Brazil; ^3^Faculdade de Farmácia e Centro de Biotecnologia, Universidade Federal do Rio Grande do Sul, Avenida Ipiranga, CEP 90610-000 Porto Alegre, RS, Brazil; ^4^Laboratório de Genética de Microrganismos, Departamento de Antibióticos, Centro de Ciências Biológicas, Universidade Federal de Pernambuco, Avenida Professor Moraes Rêgo, s/n Cidade Universitária, 50670-420 Recife, PE, Brazil

## Abstract

This study evaluated the antimicrobial and hemolytic activities and phytochemical constituents of hydroalcoholic extract and its fractions from *Buchenavia tetraphylla* leaves. Cyclohexane (BTCF), ethyl acetate (BTEF), and n-butanol-soluble (BTSBF) and non-soluble (BTNBF) fractions were obtained from a liquid-liquid partition of hydroalcoholic extract (BTHE) from *B. tetraphylla* leaves. The hemolytic activity of active fractions was checked. The BTHE inhibited the growth of *Micrococcus luteus* (MIC: 0.10 mg/mL), *Pseudomonas aeruginosa* (MIC: 0.20 mg/mL), *Mycobacterium smegmatis* (MIC: 0.39 mg/mL), *Proteus vulgaris,* and *Staphylococcus aureus* (MIC: 0.78 mg/mL for both). The more active fractions were BTCF and BTBSF. BTCF showed better potential to inhibit *M. luteus* (0.10 mg/mL), *P. aeruginosa* (0.20 mg/mL), *S. enteritidis* (0.39 mg/mL), and *S. aureus* (1.56 mg/mL). BTBSF showed the best results for *M. luteus* (0.10 mg/mL), *M. smegmatis*, *B. subtilis* (0.39 mg/mL for both), and *P. vulgaris* (0.10 mg/mL). The HC50 were greater than observed MIC: 20.30, 4.70 and 2.53 mg/mL, respectively, to BTBF, BTHE and BTCF, which. The phytochemical analysis detected the presence of flavanoids, triterpene, carbohydrate, and tannin. Our work showed for the first time the broad-spread antimicrobial activity of *B. tetraphylla*, which has nonhemolytic action, creating a new perspective on the interesting association of traditional and scientific knowledge.

## 1. Introduction

Traditional medicine is used by a large proportion of the semiarid Brazilian population as the major health need of humans and animals [1, 2]. Caatinga medicinal plants have become the focus of intense study recently in terms of conservation and as to whether their traditional uses are supported by actual pharmacological effects or merely folklore [3, 4]. With the increasing acceptance of herbal medicine as an alternative form of health care, the screening of Caatinga medicinal plants for bioactive compounds is important and has been confirmed by the traditional uses [5–9].

In this context, many species of the Combretaceae are used medicinally in several continents in the world. In northeastern Brazil, Agra et al. [10] listed many more traditional medicinal uses of the Combretaceae, which include anthelminthic, treatment of acute enteritis, colitis, constipation, dental caries, diuretic, inflammations in general, malaria, tuberculosis, and cancer, among others. 

*Buchenavia* is a genus of Combretaceae family comprising about 25 species distributed on Central America (Cuba, Trinidad, Panama, and West Indies), Venezuela, Colombia, Guyana, Brazil, Peru, and Bolivia. In the Amazon region, there is the highest concentration of species (20), six occur in the southeast and one reaches the southern Brazil (Santa Catarina). *Buchenavia tetraphylla* (Aubl.) R. A. Howard (Combretaceae: Combretoideae) is a neotropical species with distribution from Cuba Island (Central America) to Rio de Janeiro state, southern Brazil (South America) [11]. In Brazil this plant is known as “tanimbuca” and it is related as an ethnomedicinal plant by traditional communities in the region northeast of Brazil, including indigenous groups [4, 12]. An anti-HIV alkaloid was previously isolated from the leaves of this plant but its cytotoxicity led to a lower therapeutic index [13].

In this work we performed a phytochemical screening of *B. tetraphylla* leaves, examined the antimicrobial activity of hydroalcoholic crude extract and its fractions, and checked the hemolytic effect of more active samples.

## 2. Materials and Methods

### 2.1. Plant Collection and Plant Storage

Leaves of *B. tetraphylla* were collected *in Parque Nacional do Catimbau, *Pernambuco, Brazil, northeastern Brazil, in September 2010. Botanical identification was made by staff of the herbarium of Instituto de Pesquisa Agronômica de Pernambuco (IPA), Brazil, and voucher specimens were deposited in the herbarium (IPA 84.104). Leaves were dried at room temperature. The dried plants were milled to a fine powder in a Macsalab mill (Model 200 LAB), Eriez, Bramley, and stored at room temperature in closed containers in the dark until used.

### 2.2. Preparation of the Crude Hydroalcoholic Extract

*B. tetraphylla* leaves were dried at room temperature for 7 days, ground into a fine powder and used for extraction. The powder (20 g) was mixed with 50 mL ethanol : water (7 : 3) and submitted to agitation for 15 hours. Then the extracts were filtered and the powder residue was mixed again with 50 mL ethanol-water and the entire extraction process was repeated. The supernatants collected were mixed in a round bottom flask and concentrated at 45°C. The residue was dissolved in DMSO (dimethyl sulfoxide) and kept at −20°C until use.

### 2.3. Phytochemical Analysis

The phytochemical tests to detect the presence of tannins, flavonoids, anthocyanins, saponins, coumarins, quinones, anthraquinones, reducers compounds, and alkaloids were performed according to the method described by Kokate [14] and Harborne [15].

### 2.4. Fractionation of the Hydroalcoholic Extract

The hydroalcoholic extract was dissolved in water, producing a solution that was submitted to liquid-liquid partitions successively with cyclohexane, ethyl acetate, and n-butanol. The solutions produced were dried in anhydrous Na_2_SO_4_ and submitted to filtration under reduced pressure. Thereafter, the solvents were evaporated under reduced pressure in a rotary evaporator oven at 60°C, producing hexane, ethyl acetate, n-butanol soluble, and n-butanol nonsoluble phases. The residues obtained were kept at −20°C for future use.

### 2.5. Microbial Strains

The antimicrobial activity of *B. tetraphylla* leaves extract and its fractions were tested against the following microorganisms: *Staphylococcus aureus* (UFPEDA02), *Mycobacterium smegmatis* (UFPEDA71), *Bacillus subtilis* (UFPEDA82), *Micrococcus luteus* (UFPEDA100), *Enterococcus faecalis* (UFPEDA138), *Escherichia coli* (UFPEDA 224), *Klebsiella pneumoniae* (UFPEDA 396), *Salmonella enteritidis* (UFPEDA 414), *Pseudomonas aeruginosa* (UFPEDA416), *Proteus vulgaris* (UFPEDA740), *Candida krusei* (UFPEDA1002), *Candida albicans* (UFPEDA1007), and *Aspergillus niger* (UFPEDA2003). All strains were provided by Departamento de Antibióticos, Universidade Federal de Pernambuco (UFPEDA) (Table 1) and maintained in Nutrient Agar (NA) and stored at 4°C.

### 2.6. Determination of Antibacterial Activity Using the Disc Diffusion Method

The antibacterial activity of the extracts was determined by the disc diffusion method [16]. Briefly, bacterial strains were grown on Mueller-Hinton Agar (MHA) medium at 37°C for 18 hours, suspended in distillated water (approximately 1.5 × 10^8^ CFU/mL). An aliquot of 100 *μ*L of bacterial suspension was immediately inoculated in petri dishes containing MHA medium. Sterile paper discs containing 2000 *μ*g of extracts were added to the culture plates and the samples were incubated at 37°C for an additional 18 hours. After incubation, the diameter of the zone of growth inhibition was examined. Antibiotics and DMSO were used as the negative control.

### 2.7. Minimum Inhibitory Concentration and the Minimum Bactericidal Concentration

Minimum inhibitory concentration (MIC) was determined by the microdilution method [17]. A twofold serial dilution of the extract/fractions was prepared in Mueller Hinton Broth (MHB) and 100 *μ*L (approximately 1.5 × 10^8^ CFU/mL) of bacteria suspension was added. The samples were incubated for 24 h at 37°C. Resazurin solution (0.01%) was used as an indicator by color change visualization: any color changes from purple to pink were recorded as bacterial growth. The lowest concentration at which no color change occurred was taken as the MIC. Afterwards, cultures were seeded in MHA medium and incubated for 24 h at 37°C to determine the minimum bactericidal concentration (MBC) which corresponds to the minimum concentration of extract/fractions that eliminated the bacteria.

### 2.8. *In Vitro* Hemolytic Assay

Blood (5–10 mL) was obtained from healthy nonsmoking volunteers by venipuncture, after a written informed consent was obtained. Human erythrocytes from citrated blood were immediately isolated by centrifugation at 1500 rpm for 10 min at 4°C. After removal of plasma and buffy coat, the erythrocytes were washed three times with phosphate-buffered saline (PBS; pH 7.4) and then resuspended using the same buffer and a 1% erythrocyte suspension was prepared. The hemolytic activity of the crude extract was tested under *in vitro* conditions. Each tube received 1.1 mL of erythrocyte suspension and 0.4 mL of extract of various concentrations (50–500 *μ*g/mL) were added. The negative control was only solvent and the positive control received 0.4 mL of Quillaja saponin (0.0025%). After 60-min incubation at room temperature, cells were centrifuged and the supernatant was used to measure the absorbance of the liberated hemoglobin at 540 nm. The average value was calculated from triplicate assays. The hemolytic activity was expressed in relation to ascorbic acid and calculated by the following formula [18]:

(1) $$\begin{array}{c} \text{hemolytic}\;\;\text{activity}\;\;\left(\%\right)=\frac{\left(A_{s}-A_{b}\right)}{\left(A_{c}-A_{b}\right)}\times 100, \end{array}$$

where *A*_*c*_ was the absorbance of the control (blank, without extract), *A*_*s*_ was the absorbance in the presence of the extract, and *A*_*c*_ was the absorbance of saponin solution.

### 2.9. Statistical Analysis

Each experiment was performed in triplicate and results are expressed as the mean ± SD (standard deviation). Statistical analysis was performed by Student's *t*-test. Differences were considered significant at *P* < 0.05.

## 3. Results and Discussion

The results from the present study showed that at least one of BTHE and its fractions displayed antimicrobial activities against all the pathogens tested, except for *A. niger* (Table 1). However, the inhibition varied according to extract/fractions and microorganism tested. In addition, the extract and its fractions exhibited broad spectrum of activity.

The BTHE showed inhibition diameter zones (IDZs) ranging from 0 to 27 mm, with the highest IDZs observed against *M. smegmatis* and *M. luteus* (27 mm), followed by *S. aureus* (21.70 mm), *P. aeruginosa* (18.5 mm), *P. vulgaris* (18 mm), *S. enteritidis* (12 mm), and *B. subtilis *(9.7 mm). The IDZs against two yeasts were in a partial way with IDZs of 17.7 and 22 mm to *C. krusei* and *C. albicans*. This extract did not show activity against *E. faecalis*,* E. coli, K. pneumaniae,* and *A. niger* in disc paper assay. 

In this context, BTHE inhibited strongly the growth of *M. luteus* (MIC: 0.10 mg/mL), *P. aeruginosa* (MIC: 0.20 mg/mL), *M. smegmatis* (MIC: 0.39 mg/mL)*, P. vulgaris,* and *S. aureus* (MIC: 0.78 mg/mL for both). Antimicrobial substances are considered as bacteriostatic agents when the ratio MBC/MIC > 4 and bactericidal agents when the ratio MBC/MIC ≤ 4 [19]. Thus, BHTE was a bacteriostatic agent for these pathogens. For the other pathogens the MIC values were greater than 1 mg/mL.

In relation to antimicrobial activities of fractions, the best results were found in cyclohexane (BTCF) and n-butanol soluble fractions (BTBSF), followed by n-butanol non-soluble (BTNBF) and ethyl acetate fractions (BTEF). The most active fraction was BTCF which showed better potential (MIC < 1 mg/mL) to inhibit the growth of *M. luteus* (MIC: 0.10 mg/mL), *P. aeruginosa* (MIC: 0.20 mg/mL), *S. enteritidis* (MIC: 0.39 mg/mL), and *S. aureus* (MIC: 1.56 mg/mL). The BTBSF showed the best results for *M. luteus *(0.10 mg/mL), *M. smegmatis*, *B. subtilis* (0.39 mg/mL for both), and *P. vulgaris* (0.10 mg/mL) (Table 1). 

The results of phytochemical screening of *B. tetraphylla* leaves showed the presence of flavanoids (luteolin), proanthocyanidin, leucoanthocianidin, triterpene, Carbohydrate, and Gallic Tannin. Our results revealed that all of the fractions showed antimicrobial activity suggesting that all solvents are able to solubilize at least one kind of active compounds.

Flavonoids are ubiquitous in photosynthesizing cells and therefore occur widely in plant kingdom [20]. The antibacterial activity of flavonoids has been documented in several earlier studies [21, 22]. Flavonoids have multiple cellular targets and may act as nucleic acid synthesis, cytoplasmic membrane function, or energy metabolism inhibitor. Also, flavonoids are bacteriostatic compounds which induce the formation of bacterial aggregates thereby reducing the number of viable colonies [23]. The presence of luteolin, which has a hydroxyl group at the 3′ position, was detected, being known as a powerful antimicrobial agent [21]. The proantocyanidin have showed ability to protect the urinary tract infections and antioxidant activity [24]. 

Terpenoids are the largest and the most diverse class of plant compounds and they have numerous functional roles in metabolism and in ecological interactions [25]. These products are soluble in nonpolar solvent and have been showed a lot of biotechnologic activity and were found in *B. tetraphylla* leaves. Terpenes are active against bacteria and fungi [26].

Another compound detected in our study was the gallic tannin, which belongs to the tannins class, a group of polymeric phenolic substances capable of tanning leather or precipitating gelatin from solution, a property known as astringency, commonly found in higher herbaceous and woody plants. Many human physiological activities, such as stimulation of phagocytic cells, host-mediated tumor activity, and a wide range of anti-infective actions, have been assigned to tannin. Their mode of antimicrobial action, as described in the section on quinones, may be related to their ability to inactivate microbial adhesins, enzymes, cell envelope transport proteins, and so forth [27].

Cellular toxicity of the extract and most active fractions (BTBSF, BTCF) was also evaluated using human erythrocytes as a test system. These extract and fractions showed HC50 (the concentration needed for 50% of hemolysis) of 20.30, 4.70, and 2.53 mg/mL, respectively, to BTBF, BTHE, and BTCF. It is important to note that these concentrations are much lower than MIC values.

In conclusion, our work showed that *B. tetraphylla* leaves have antimicrobial activity in a broad-spread way. This plant was able to inhibit strongly the growth of *S. aureus*, *B. subtilis*, *S. enteritidis*, *M. smegmatis*, *M. luteus,* and *P. aeruginosa*. The active extract and fractions did not show hemolytic activity at MIC values, advocating thereby their safety in therapeutic use. To the best of our knowledge this is the first paper about antimicrobial activity of *B. tetraphylla*. The isolation and chemical characterization of these extracts are being performed by our group and represent a sustainable possibility to the utilization of the natural resources from Caatinga.

## Figures and Tables

**Table 1:** Antimicrobial activity of *Buchenavia tetraphylla* leaves.

Microorganism^1^	BTHE				BTCF				BTEF				BTBSF				BTBNF			
	IDZ^2^	MIC^3^	MBC^3^	MBC/MIC	IDZ	MIC	MBC	MBC/MIC	IDZ	MIC	MBC	MBC/MIC	IDZ	MIC	MBC	MBC/MIC	IDZ	MIC	MBC	MBC/MIC
02	21.70	0.78	3.13	4	24.00	1.56	3.13	2	27.70	3.13	12.50	4	24.70	3.13	6.25	2	26.00	6.25	12.50	2
71	27.00	0.39	6.25	16	27.00	0.78	3.13	4	29.00	0.78	6.25	8	26.00	0.39	3.13	8	30.00	0.78	12.50	16
82	28.70	0.39	0.78	2	29.00	0.20	0.78	4	33.00	0.78	1.56	2	28.00	0.39	0.78	2	29.00	0.78	6.25	8
100	27.00	0.10	0.39	4	30.00	0.10	0.20	2	34.00	0.20	1.56	8	29.00	0.10	0.39	4	31.00	0.20	1.56	8
138	0.00	25.00	>25	>1	0.00	25.00	>25	>1	0.00	25.00	>25	>1	0.00	12.50	25.00	2	0.00	25.00	25.00	1
224	0.00	6.25	>25	>4	0.00	3.13	25.00	8	0.00	6.25	12.50	2	19.70	3.13	25.00	8	0.00	12.50	25.00	2
396	0.00	12.50	25.0	2	18.00	6.25	12.50	2	0.00	12.50	25.00	2	19.00	12.50	12.50	1	19.30	12.50	12.50	1
414	12.00	0.78	12.5	16	13.00	0.39	0.78	2	15.00	0.78	1.56	2	13.00	0.78	0.78	1	14.00	1.56	3.13	2
416	18.50	0.20	1.56	8	21.00	0.20	0.78	4	24.00	0.20	3.13	16	22.00	0.20	1.56	8	22.70	0.78	3.13	4
740	18.00	0.78	1.56	2	20.00	0.39	1.56	4	23.00	0.39	3.13	8	21.00	0.10	0.78	8	25.00	0.78	6.25	8
1002	17.7	6.25	25.0	4	19.00	3.13	12.50	4	21.00	3.13	25.00	8	20.00	6.25	12.50	2	21.00	6.25	12.50	2
1007	22	12.50	>25	>2	25.00	12.50	25.00	2	28.00	12.50	25.00	2	22.00	12.50	25.00	2	24.00	12.50	25.00	2
2003	0.00	25.00	>25	>1	0.00	25.00	>25	>1	0.00	25.00	>25	>1	0.00	25.00	>25	>1	0.00	25.00	>25	>1

^1^ *Staphylococcus  aureus *(UFPEDA 02), *Mycobacterium  smegmatis *(UFPEDA 71), *Bacillus subtilis* (UFPEDA 82), *Micrococcus luteus* (UFPEDA 100), *Enterococcus faecalis* (UFPEDA 138), *Escherichia coli* (UFPEDA 224), *Klebsiella pneumaniae* (UFPEDA 396), *Salmonella enteritidis *(UFPEDA 414), *Pseudomonas aeruginosa *(UFPEDA 416), *Proteus vulgaris *(UFPEDA 740), *Candida  krusei* (UFPEDA 1002), and *Candida albicans* (UFPEDA 1007).

^2^IDZ is expressed in mm.

^3^MIC and MMC are expressed in mg/mL.
